# Does digital technology make people healthier: the impact of digital use on the lifestyle of Chinese older adults

**DOI:** 10.1186/s12877-023-04651-1

**Published:** 2024-01-22

**Authors:** Kaichang Cui, Wei Zou, Xiang Ji, Xinghui Zhang

**Affiliations:** 1 Social Security Research Center, Shanghai University of Engineering Science, Shanghai, China; 2 School of Management, Shanghai University of Engineering Science, Shanghai, China

**Keywords:** China, Digital use, Older adult, Healthy lifestyle, Structural equation model

## Abstract

**Background:**

With the arrival of the era of large-scale production, sharing and application of data, digital use has gradually changed people’s daily entertainment, consumption, social interaction, learning and other behaviors in its efficient form. This paper mainly discusses whether this fast and convenient behavior leads Chinese older adults to adopt healthier lifestyles.

**Methods:**

Using the most recent information from the Chinese Family Panel Studies (CFPS) in 2020, this paper conducted a descriptive statistical analysis on the basic situation of digital use and lifestyles among Chinese older adults and used a structural equation model to analyse the influence of frequency and types of digital use in a variety of different aspects of the real life of Chinese older adults.

**Results:**

Research revealed that the quality of life of Chinese older adults improved significantly as a result of their use of digital technology. The frequency of digital use (FDU) significantly improved Chinese older adults’ diet, sleep, exercise, smoking and drinking, and relieved their depression. The types of digital use (TDU) had a significant positive correlation with the lifestyle of Chinese older adults, especially in the influence of digital entertainment (DE), digital consumption (DC) and digital social interaction (DI) on the lifestyle of Chinese older adults.

**Conclusions:**

Digital use can improve the health of Chinese older adults by promoting a healthy lifestyle through various means. The findings of this study have a substantial positive impact on bridging the digital divide that Chinese older adults face, as well as fostering the integration of digital use into their healthy lifestyles.

## Background

The digital economy has brought about significant changes in social production and lifestyle, facilitating more convenient and streamlined lifestyles for individuals. Based on “The 52nd Statistical Report on China’s Internet Development” [1], the total number of Internet users in China has reached a staggering 1.08 billion individuals, with an adoption rate exceeding 80% for Internet applications, such as instant messaging, online video streaming, short video, online payment services, and online purchasing among Internet users. However, the degree of Chinese population ageing has been at the upper middle level compared to global standards, and the trend of ageing is increasingly prominent. According to the Seventh National Census, Chinese population aged 60 and above stood at 264.019 million as of the conclusion of 2020, constituting approximately 18.70% of the overall population. During China’s 14th Five-Year Plan (2021–2025), the population of Chinese older adults is projected to exceed 300 million, transitioning from a stage of mild ageing to one of moderate ageing. Consequently, there is a growing need for adopting and maintaining a health-promoting lifestyle in Chinese older adults.

A healthy lifestyle encompasses spontaneous and multifaceted behaviors and perceptions of individuals, aiming to achieve self-satisfaction in maintaining or improving health status [2]. The “Basic Knowledge and Skills of Health Literacy of Chinese Citizens” [3], issued by the National Health Commission of the PRC, defines a healthy lifestyle based on four aspects: reasonable diet, moderate physical activity, smoking cessation and alcohol reduction, and mental balance. Additionally, sleep is also taken as one of the criteria for measuring a healthy lifestyle that can effectively prevent or delay the onset of various chronic diseases, exerting a significant impact on disease morbidity and mortality [4]. Several studies indicated that digital use is closely related to older adults’ healthy lifestyle, including physical exercise [5] and mental health [6]. Digital use not only ensures that older adults’ basic requirements are met in the information age but also serves as a vital means to improve individuals’ well-being and enrich their over life experience [7]. Concurrently, the research indicated that digital use can facilitate older adults in broadening their access to health information and protecting their own rights and interests, thereby guiding them towards adopting a wholesome lifestyle [8].

Prior research has primarily addressed the digital divide and the effect of digital use on the psychological [9, 10] and physical well-being [7, 11] of older adults. However, there is limited scholarly work directly linking digital use with a healthy lifestyle among Chinese older adults. Broadly, lifestyle has been used as a mediator in examining the impacts of digital use on older adults’ psychological well-being [12], engagement in society [13] and subjective well-being [14]. From a current perspective, this paper examines the potential for digital use to enhance the health of Chinese older adults. It carefully selects relevant variables to gain insights into the current basic situation of digital use and healthy lifestyle among Chinese older adults. Furthermore, it delves into the association between digital use, including frequency and types, and lifestyle, aiming to establish a well-rounded lifestyle for Chinese older adults.

Regarding the impact of digital use on the healthy lifestyle of Chinese older adults, there are two theoretical perspectives from the lens of Internet use. One perspective suggests that the Internet possesses an augmenting effect and positively influences the lifestyle of older adults. Wallinheimo et al. [15] suggested that people with a superior quality of life prefer to use the Internet to communicate and quality of life is inseparable from lifestyle [16]. Additionally, Quittschalle et al. [17] argued that the Internet has the potential in promoting a healthy lifestyle, and Cui et al. [18] found a strong association between e-health literacy and healthy behaviors among older adults. The other perspective suggests that the Internet possesses a substitution effect, which is not conducive to the formation of a healthy lifestyle for older adults. Social withdrawal and impaired psychological wellbeing, such as depression and loneliness, invariably come with Internet addiction [19, 20], ultimately leading to an unhealthy lifestyle. In conclusion, this study proposes the first hypothesis:

### H1

More frequent digital use is associated with a healthier lifestyle among Chinese older adults.

Researchers analysed the association between digital use and lifestyle based on various aspects, including diet, exercise, sleep, smoking and drinking, as well as depression. Sun et al. [21] surveyed using questionnaires and found that the health information accessed by older adults on the Internet is related to diet and physical activity. Zhang et al. [12] discovered a statistically significant positive association between digital use among older adults and their mental well-being, with physical exercise mediating in this relationship. Firth et al. [22] investigated the relationship between lifestyle factors such as exercise, smoking, nutrition, sleep and mental health, and found that a healthy lifestyle is conducive to positive psychology well-being, as well as the potential impact of digital technology on mental health. Drawing from the existing literature, this study puts forth a set of five sub-hypotheses:

### H1-1

Frequency of digital use (FDU) is associated with diet among Chinese older adults.

### H1-2

FDU is associated with exercise among Chinese older adults.

### H1-3

FDU is associated with sleep among Chinese older adults.

### H1-4

FDU is associated with smoking and drinking among Chinese older adults.

### H1-5

FDU is associated with depression among Chinese older adults.

**Fig. 1:**
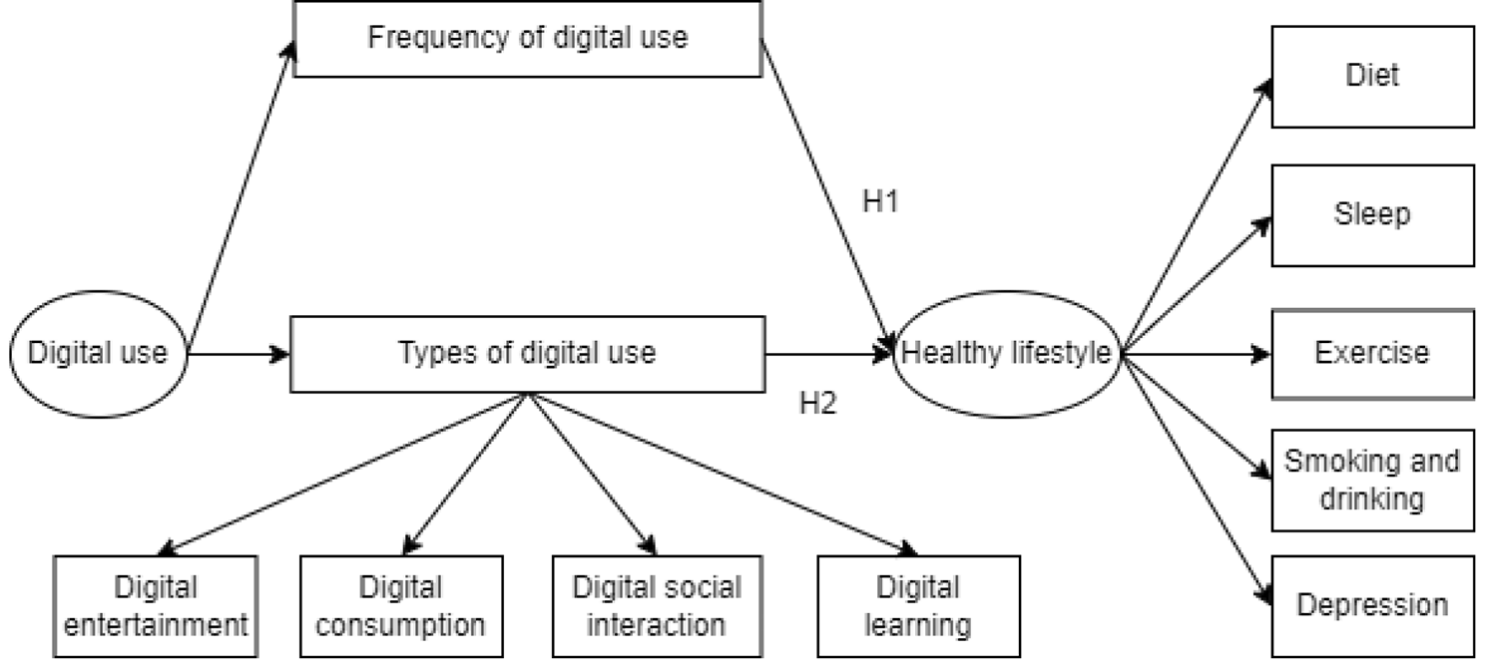
The theoretical framework of the study

Type and frequency are two fundamental aspects of digital use. Regarding the types of digital use, existing researches primarily focus on investigating digital entertainment, digital consumption, digital social interaction and digital learning as they relate to older adults’ lifestyles. Concerning digital entertainment, virtual reality games have the potential to enhance physical abilities among older adults [23], as well as improve memory associated with the hippocampus in a population already facing age-related cognitive decline [24]. Simultaneously, the rise of the Internet and e-commerce has attracted older adults to try online shopping, and consumer culture subtly affects the consumption motivation and behavior of older adults [25]. In addition, studies indicated that engaging in digital social interaction is beneficial for older adults’ participation in community activities and the expansion of social networks [26]. In the terms of digital learning, it can promote their learning motivation and provide a solid foundation for the lifelong learning of older adults [27]. In summary, digital use has the potential to enrich the lifestyle of Chinese older adults, consequently impacting their overall physical and mental health. Therefore, this study proposes the final hypothesis (Fig. 1):

### H2

Different types of digital use (TDU) are associated with the lifestyles of Chinese older adults.

## Methods

### Data and study sample

The research utilized data from the 2020 Chinese Family Panel Studies (CFPS). The baseline sample of the CFPS encompassed 25 provinces/municipalities/autonomous regions, ensuring highly representative national population coverage. The CFPS collected data from three dimensions: individuals, households, and communities, using a variety of questionnaires in the form of lengthy interviews, short interviews, return visits, and telephone interviews. Its primary emphasis was on the well-being of Chinese inhabitants, encompassing various areas of study including economic engagement, educational achievements, familial connections and dynamics, population movement, and overall health. To ensure the currency and validity of the results, the most recent survey data from the CFPS 2020 were employed in this study. The study population of this paper was 60 years old and above. Sample screening was performed by looking for age variables in the questionnaire, screening out samples younger than 60 years, and eliminating samples with incomplete values in the dependent, independent, and control variables from the rest of the sample. Finally, the count of samples obtained was 4162, out of which the sample size for Chinese older adults who had used the Internet amounts to 907.

### Core explanatory variables

Measuring the frequency of digital use (FDU) and types of digital use (TDU) among Chinese older adults can more accurately and comprehensively observe the current situation and existing problems associated with digital use and reflect their digital ability. According to the Chinese government issued solution to older adults’ digital use difficulties, FDU was measured by mobile device and computer usage, and TDU was subdivided into four dimensions: digital entertainment (DE), digital consumption (DC), digital social interaction (DI) and digital learning (DL). DE included Chinese older adults playing online games and watching short videos, DC reflected Chinese older adults’ ability to purchase goods over the Internet, DI focused on their utilization of social software for communicating with family and friends online, and DL assessed their engagement in electronic-based learning. The corresponding metrics for each dimension are shown in Table 1.

**Table 1:** Measured indicators and definitions

Variables		Variable Definitions
Frequency of digital use (FDU)		The substitute variable for frequency of digital use considered 1) online duration of mobile devices (scale: 0–18); 2) online duration of computer use (scale: 0–10); 3) whether to use online computer (1 = yes, 0 = no).
Types of digital use (TDU)	Digital entertainment (DE)	The substitute variable for digital entertainment considered 1) whether to play online games (1 = yes, 0 = no); 2) frequency of playing online games (never, 0; not every day, 1; daily, 2); 3) whether to watch a short video (1 = yes, 0 = no); 4) frequency of watching the short video (never, 0; not every day, 1; daily, 2); 5) the importance of the Internet to leisure and entertainment (scale: 1–5).
	Digital consumption (DC)	The substitute variable for digital consumption considered 1) whether to shop online (1 = yes, 0 = no); 2) frequency of online shopping (never, 0; not every day, 1; daily, 2); 3) the importance of the Internet in daily life (scale: 1–5).
	Digital social interaction (DI)	The substitute variable for digital social interaction considered 1) whether to use WeChat (1 = yes, 0 = no); 2) frequency of sharing moments with friends online (never, 0; not every day, 1; daily, 2); 3) the importance of the Internet for social interaction (scale: 1–5).
	Digital learning (DL)	The substitute variable for digital learning considered 1) whether to learn online every day (1 = yes, 0 = no); 2) frequency of online learning (never, 0; not every day, 1; daily,2); 3) the importance of online learning (scale: 1–5).
Diet		The substitute variable for diet considered 1) whether to consume protein in the past week (1 = yes, 0 = no); 2) whether to consume fruits and vegetables in the past week (1 = yes, 0 = no). The closer to 1, the healthier.
Sleep		The substitute variable for sleep considered 1) sleep duration (sleep ≤ 5 h or sleep ≥ 9 h, 0; 5 h<sleep<9 h, 1); 2) whether to take a afternoon nap (1 = yes, 0 = no). The closer to 1, the healthier.
Exercise		The substitute variable for exercise considered 1) exercise frequency (never, 1; less than once per month, 2; more than once per month but less than once per week, 3; 1–2 times per week, 4; 3–4 times per week, 5; more than 5 times per week, 6); 2) exercise duration (0, 0 min; 1, 1-60 min; 2 61-120 min; 3, duration over120min); 3) exercise feeling (1, feeling bad; 2, feeling ordinary; 3, feeling good). The higher the number, the healthier.
Smoking and drinking		The substitute variable for smoking and drinking considered 1) number of cigarettes smoked per day (0, do not smoke; 1, smoke 1–20; 2, smoke 21–40; 3, smoke over 40); 2) whether to smoke in the past month (1 = yes, 0 = no); 3) whether to drink alcohol more than three times in the past (1 = yes, 0 = no). The lower the number, the healthier.
Depression		The depression variable was measured by the Center for the Epidemiological Studies Depression Scale (CES-D), which comprises of eight items: 1) I feel depressed; 2) I find it difficult to do anything; 3) My sleep is not good; 4) I feel happy; 5) I experience a sense of solitude; 6) I have a happy life; 7) I feel sad; 8) I do not think life can go on (1, never; 2, sometimes; 3, often; 4, always). The lower the number, the healthier.
Gender		0, female; 1, male
Age		1, 60–69 years; 2, 70–79 years; 3, 80 years and older
Area		0, rural area; 1, urban area
Participation on medical insurance		1 = yes, 0 = no
Education		0, uneducated; 1, educated (at least primary school)

### Explained variables

The explained variables in this paper were based on the exploration of the health literacy of Chinese citizens in the “Basic Knowledge and Skills of Health Literacy among Chinese Citizens”. According to the official document, the lifestyles of Chinese older adults can be categorized into five distinct classifications: diet, sleep, exercise, smoking and drinking, and depression level. Based on the CFPS data, diet was assessed by intake of protein, fruits and vegetables. Increasing protein, fruit and vegetables in older adults elicits health benefits [28, 29]. Sleep was evaluated by sleep duration and afternoon nap habit. Afternoon nap is the prevailing break during the workday, exerting an impact on individuals’ emotional states and expressive behaviors [30]. And irregular breaks, whether too long or too short, are indicative of an unhealthy lifestyle [31, 32]. Regarding exercise in older adults, most existing studies focused on physical exercise measured by time, frequency and intensity [33]. Therefore, this paper selected three variables of exercise frequency, exercise duration and exercise feeling to measure, and higher levels of physical activity corresponded to a greater likelihood of adopting a healthy lifestyle [34]. Smoking and drinking was measured by the frequency of consumption of tobacco and alcohol, which may exhibit a negative correlation with the adoption of a healthy lifestyle [35]. The depression level of Chinese older adults was evaluated by the Center for the Epidemiological Studies Depression Scale (CES-D), which is often linked to people’s mental health status according to the CFPS [36]. The corresponding metrics for each dimension are shown in Table 1.

### Control variables

This research made reference to the existing literature [37] and identified control variables from three dimensions: individual, economic characteristic, and regional aspects. Specifically, selected control variables for this study included gender, age, area, education level, and participation in medical insurance (Table 1). Firstly, age and gender are basic demographic elements [38]. Secondly, education level reflects the socioeconomic status of older adults from one side and is closely related to their lifestyle and network participation [39]. Thirdly, regional characteristics are mainly decided whether older adults live in the city or the village, which makes a difference in the frequency and content of digital use among urban and rural older adults [40]. Finally, measuring the participation of older adults in medical insurance can intuitively reflect their physical condition and economic ability [41], thus better serving the research topic of this paper.

### Statistical analysis

Firstly, using t-test, cross-tabulation analysis and chi-squared test, the descriptive statistical analysis was employed to characterize the fundamental state of digital use among Chinese older adults, including breadth of digital use, FDU and TDU. Secondly, the structural equation model was applied to investigate how FDU and TDU impacted the lifestyle of Chinese older adults separately. Compared to alternative statistical approaches, the structural equation model provides a unique opportunity to investigate complex connections among numerous independent and dependent variables, and it allows for differentiation between direct, indirect, and overall effects while also illustrating indirect effects through intervening or mediating variables [42]. The structural equation model employs three methods of model parameter estimation: CB-SEM, PLS-SEM, and PLSc [43]. Among these methods, CB-SEM is the most prevalent and effectively minimizes the difference between the sample variance–covariance matrix and the model’s expected values to derive accurate parameter estimates [44], which have been employed in this study. The KMO and Barlett’s test can test the adequacy of the correlation matrix relationship, thereby determining whether a validity analysis should be performed. In this paper, the KMO and Barlett’s test were performed on the indicators of the lifestyle variable. The KMO was 0.835, and the significance of Bartlett’s tests was approximately 0.000 (< 0.001). Therefore, it could be concluded that the constructed index system was suitable for validity analysis. Through the exploratory factor analysis of samples, Principal Component Analysis was used to extract factors, and the cumulative variance contribution rate reached 72.799%, indicating that it could fully reflect the original data. Orthogonal rotation was used to extract the cofactors, and the factor composition was consistent with the index construction proposed in this paper, so it had a high degree of validity. Based on this, two structural equation models were developed in this study. Model 1 investigated the influence of FDU on the lifestyle of Chinese older adults, and the overall level of fit was good (RMSEA = 0.041, CFI = 0.949, TLI = 0.940). Model 2 examined the impact of TDU on their lifestyle, and its overall fitting quality was also high (RMSEA = 0.071, CFI = 0.923, TLI = 0.910).

## Results

### Basic situation of digital use in Chinese older adults

#### Breadth of digital use in Chinese older adults

The breadth of digital use among Chinese older adults was reflected in the number of TDU (including DE, DC, DI and DL). The selected population of 4162 seniors used an average of 0.43 TDU, while 79.1% never used any digital types. According to the t-test results (Table 2), the number of TDU by males was 0.09 greater than that of women, highlighting the unfairness of healthy ageing in elderly females. In addition, the number of TDU by Chinese older adults aged 60–69 years was 0.26 and 0.42 higher than those aged 70–79 years and over 80 years, respectively, indicating a noticeable disparity in the number of TDU among various age groups. The TDU count for Chinese older adults residing in urban regions was 0.42 higher than their counterparts in rural areas. Inequality in regional digital access conditions may also be an important factor influencing digital use. Finally, the number of TDU by the educated was 0.53 higher than that of the uneducated, demonstrating disparities due to differences in digital skills. The number of TDU was 0.14 higher among those with Medicare than among those without, and the number of TDU did not vary significantly based on whether they lived alone or not.

**Table 2:** Independent samples t-test of the breadth of digital use in Chinese older adults (*N* = 4162)

Variable	Groups		Number of samples	Mean of the number of TDU
Breadth of digital use in Chinese older adults	Gender	Female	2019	0.38
		Male	2143	0.47
	Age	60–69 years	2684	0.53
		70–79 years	1289	0.27
		80 years and older	189	0.11
	Area	Rural area	2156	0.22
		Urban area	2006	0.64
	Education	No	1669	0.11
		Yes	2493	0.64
	Participation on medical insurance	No	342	0.30
		Yes	3820	0.44

Furthermore, when examining the samples of 907 Chinese older adults participating in digital use, the distribution of the number participating in various TDU, including DE, DC, DL and DI, more prominently illustrated the “grey digital divide”. Specifically, nearly 30% of Chinese older adults still never used or only used a single TDU, while merely 3.42% of Chinese older adults employed all four TDU’s.

#### FDU in Chinese older adults

As depicted in Table 3, Chinese older adults possessed various characteristics in different kinds of FDU. Among the 907 Chinese older adults, nearly half of them (46.5%) watched short videos (DE) every day. Almost all Chinese older adults (86.5%) did not use the Internet to play online games (DE). In the domain of DC, the majority of Chinese older adults (74.4%) did not use mobile phones and other digital devices for online shopping and other activities, while only a small percentage (25.6%) used the Internet for consumption. In addition, nearly half of Chinese older adults (46.9%) refrained from digital devices for social interaction (DI), whereas 9.8% used digital devices daily to communicate with others. In the field of digital learning, the overwhelming majority of Chinese older adults (90.2%) did not use smart devices for learning activities such as reading books and newspapers (DL).

**Table 3:** The basic situation of FDU in Chinese older adults (%)

FDU(*N* = 907)	Never use	Not every day	Daily
DE (short video)	36.6	16.9	46.5
DE (online game)	86.5	6.3	7.2
DC	74.4	22.8	2.8
DI	46.9	43.3	9.8
DL	90.2	4.2	5.6

#### TDU in Chinese older adults

Based on cross-tabulation analysis and chi-squared test, the results of a heterogeneity analysis of the TDU in Chinese older adults are presented in Table 4. In the sample of 907, the overall level of digital use was low. More Chinese older adults used digital devices to watch short videos (DE) and participate in digital social activities (DI), accounting for 63.40% and 90.41% of 907 samples, respectively. This observation can potentially be attributed to the portability and provision of spiritually enriching information by short videos [45], while digital social interaction can mitigate the retirement-related loneliness of older adults [46]. Conversely, only a small percentage of Chinese older adults used smart devices for DE (online game), DC or DL, constituting merely 13.45%, 25.58% and 9.81%, respectively. Possible explanations for this phenomenon may lie in the higher cognitive function associated with online gaming, digital consumption and digital learning which might not align with preferences for traditional lifestyles, such as offline consumption and paper reading.

**Table 4:** Heterogeneity analysis of TDU in Chinese older adults (%)

TDU(*N* = 907)	Total	Female	Male	60–69 years	70−79years	80 years and older	Rural area	Urban area
DE (online game)	13.45	15.35	12.02	14.43	10.38	5.88	5.56^*^	16.80^*^
DE (short video)	63.40	64.45	62.60	66.62^*^	53.55^*^	35.29^*^	69.26^*^	60.91^*^
DC	25.58	24.30	26.55	27.02^*^	22.40^*^	0^*^	15.19^*^	29.98^*^
DI	90.41	92.07	89.15	90.81	89.07	88.24	84.81^*^	92.78^*^
DL	9.81	9.46	10.08	10.04	9.84	0	7.78	10.68

^*^ indicates passing the significance test at the 5% level

From the gender perspective, there were no significant variances in each TDU between Chinese older adults of different genders. However, considering age as a factor, it was found that the proportion of individuals aged 60–69 years who engaged in watching short videos (DE) or participating in DC was significantly higher compared to those aged 70–79 years and over 80 years, and age may be a significant factor affecting Chinese older adults watching short videos. Older adults have poorer cognitive abilities such as spatial ability [47], and are also faced with psychomotor coordination problems, decreased attention and memory decline, which make it difficult for them to master Internet skills. Furthermore, when examining regional disparities, it was observed that urban-dwelling Chinese older adults had significantly higher proportions engaging in DE (online game), DC and DI compared to their rural counterparts. The benefits of digital use are not equally accessible to older adults residing in rural and urban areas [40]. In addition, there was an attractive phenomenon that a higher proportion of rural older adults engaged in watching short videos (DE) compared to urban order adults and it was also worth further discussion in the future.

### Basic situation of lifestyle in Chinese older adults

Table 5 presents descriptive statistics on the lifestyle characteristics among Chinese older adults. At the dietary level, Chinese older adults who used digital devices tended to have higher dietary intake than those who did not, especially in terms of protein intake, indicating a more health-conscious lifestyle. Regarding sleep, Chinese older adults who used digital devices exhibited significantly healthier sleep duration compared to those who do not. In terms of exercise, both frequency and duration were notably higher among Chinese older adults using digital devices compared to non-users, as was the case for exercise feelings, and Chinese older adults who used digital devices were more likely to have a healthy exercise style. Concerning smoking and alcohol consumption, Chinese older adults with digital devices smoked slightly more cigarettes per day and month than those without such devices. However, with regard to alcohol consumption, there was no notable disparity observed between the two groups. Lastly, in relation to depression levels, Chinese older adults who used digital devices were more cheerful and less melancholic than those who did not use such devices, suggesting a potential protective effect against the development of depression.

**Table 5:** Basic situation of lifestyle in Chinese older adults

Variables	Do not use digital devices (*N* = 3255)	Use digital devices (*N* = 907)	Total (*N* = 4162)	Min	Max	Std. Dev.
Whether to consume protein in the past week	0.76	0.88	0.79	0	1	0.411
Whether to consume fruits and vegetables in the Past week	0.98	0.99	0.98	0	1	0.146
Sleep duration	0.70	0.81	0.73	0	1	0.445
Whether to take a afternoon nap	0.68	0.72	0.69	0	1	0.464
Exercise frequency	1.32	2.80	1.64	0	6	2.552
Exercise duration	0.31	0.67	0.38	0	3	0.635
Exercise feeling	0.43	1.00	0.56	0	3	0.927
Number of cigarettes smoked per day	0.30	0.32	0.31	0	3	0.520
Whether to smoke in the past month	0.27	0.29	0.28	0	1	0.449
Whether to drink alcohol more than three times in The past month	0.16	0.16	0.16	0	1	0.365
I feel depressed	1.74	1.64	1.72	1	4	0.848
I find it difficult to do anything	1.98	1.68	1.91	1	4	1.013
My sleep is not good	1.94	1.83	1.92	1	4	1.020
I feel happy	2.98	3.14	3.01	1	4	0.994
I feel lonely	1.54	1.38	1.51	1	4	0.849
I have a happy life	3.11	3.32	3.16	1	4	0.955
I feel sad	1.54	1.36	1.50	1	4	0.775
I do not think life can go on	1.33	1.14	1.29	1	4	0.673

### The impact of digital use on the lifestyle of Chinese older adults

#### The influence of FDU on the lifestyle of Chinese older adults

A bivariate analysis was performed for all explanatory variables and all indicators of explanatory variables, and preliminary findings indicated that digital use potentially had a significant favorable impact on Chinese older adults’ lifestyles. For example, among Chinese older adults, online duration of mobile devices was significantly associated with protein intake, sleep duration, exercise frequency, and CES-D metrics. This implied a potential correlation between prolonged use of digital devices and increased protein consumption, enhanced sleep quality, good exercise habits, reduced degree of depression, as well as the adoption of healthier lifestyles.

**Fig. 2:**
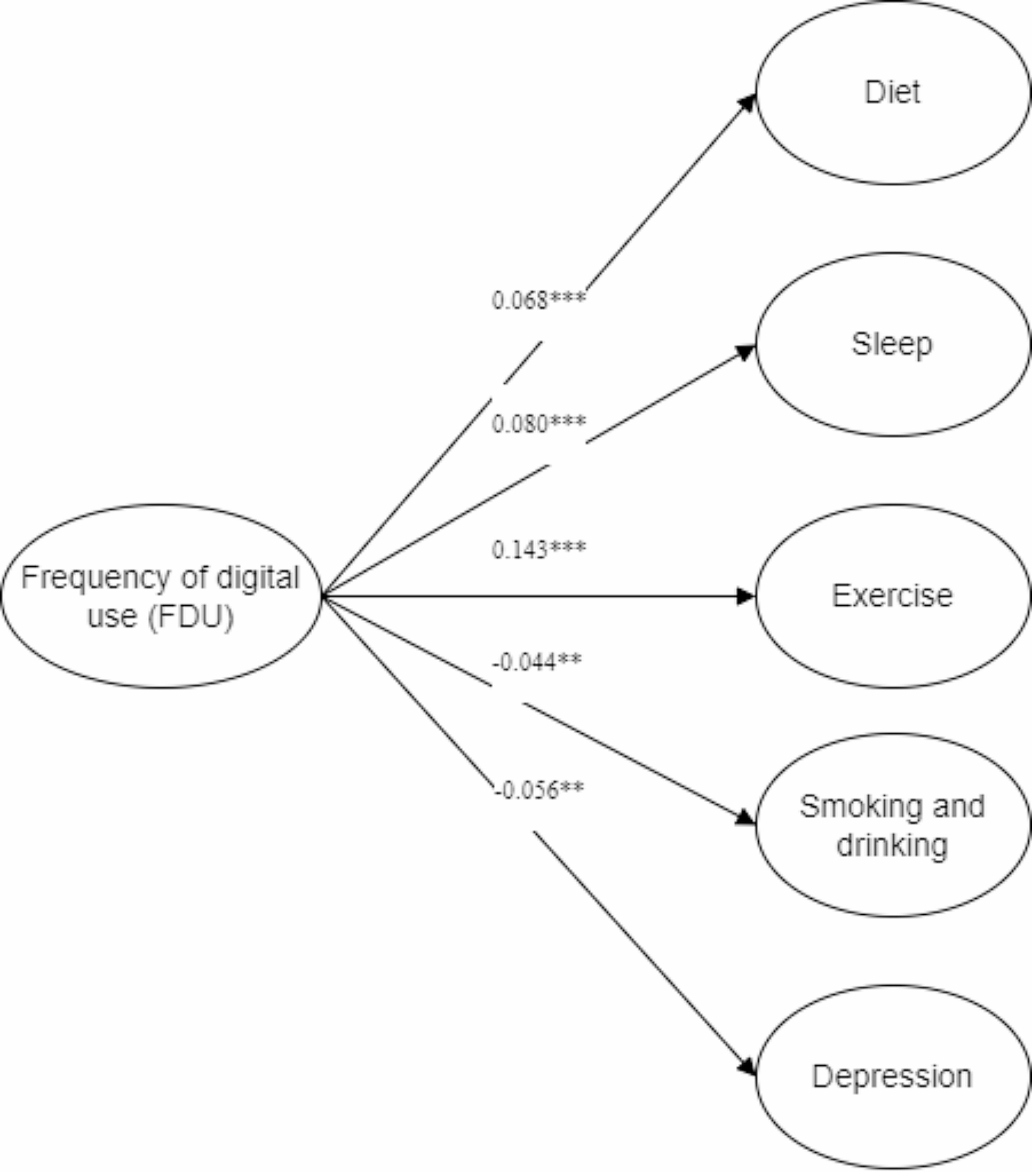
The influence path of frequency of digital use on the lifestyle of Chinese older adults. *P < 0.05, **P < 0.01, ***P < 0.001

Furthermore, the structural equation model was incorporated for analysis (Fig. 2). Model 1 examined the impact of FDU on the lifestyle of Chinese older adults and demonstrated a good fit (RMSEA = 0.041, CFI = 0.949, TLI = 0.940). After controlling for other variables, FDU was found to have a positive effect on diet, sleep, exercise, smoking and drinking, as well as depression levels among Chinese older adults. Firstly, there existed a highly significant relationship between FDU and the diet of Chinese older adults (B = 0.068, SE = 0.022, *P* < 0.001). Those who used digital devices more frequently tended to have a healthier diet. This phenomenon can potentially be attributed to the Internet serving as an essential source of health and self-care information for this demographic [48]. Chinese older adults who utilized digital devices more frequently had greater access to health-related information, thus necessitating an increased emphasis on maintaining a healthy diet. Secondly, a strong correlation was found between FDU and sleep (B = 0.080, SE = 0.025, *P* < 0.001). Chinese older adults who used digital devices more frequently tended to have better sleep duration, aligning with the findings reported by Sun [49]. Thirdly, a highly significant correlation was identified between FDU and exercise of Chinese older adults (B = 0.143, SE = 0.134, *P* < 0.001). Those who used digital devices more frequently demonstrated better exercise habits. The Internet can provide diverse health information based on individuals’ requirements [50], which in turn positively reinforces good exercise habits among older adults. Fourthly, a negative association was observed between FDU and smoking and drinking among Chinese older adults (B=-0.044, SE = 0.024, *P* = 0.003). Frequent use of digital devices was found to reduce the frequency of smoking and the likelihood of binge drinking among Chinese older adults. The integration of Internet technology has presented opportunities for older adults to connect with society and alleviate their social isolation [51], ultimately promoting healthy lifestyle habits and reducing the risk of smoking and alcohol abuse [52]. Lastly, a significant correlation was found between FDU and depression level of Chinese older adults (B=-0.056, SE = 0.030, *P* = 0.002). Increasing digital use frequency may reduce the poor depression of older adults and alleviate their loneliness [53].

#### The impact of TDU on the lifestyle of Chinese older adults

Model 2 investigated the relationship between TDU and lifestyle among Chinese older adults (Table 6), with a satisfactory fit of the model (RMSEA = 0.071, CFI = 0.923, TLI = 0.910). After controlling for other variables, various TDU had varying effects on the lifestyle among Chinese older adults. Specifically, firstly, DE significantly affected the diet (B = 0.053, SE = 0.007, *P* = 0.019), sleep (B = 0.222, SE = 0.007, *P* = 0.001), exercise (B = 0.121, SE = 0.045, *P* < 0.001) and depression (B=-0.055, SE = 0.009, *P* = 0.008) of Chinese older adults. The greater exposure to DE was associated with improved diet quality, enhanced sleep patterns and good exercise routines among Chinese older adults, while also reducing their depressive symptoms. Secondly, there was a positive correlation between DC and diet (B = 0.057, SE = 0.010, *P* = 0.006) and exercise (B = 0.109, SE = 0.058, *P* < 0.001), and a negative correlation was observed between DC and depression level (B=-0.056, SE = 0.012, *P* = 0.004). This suggests that increased exposure to DC was linked to better diet and higher engagement in physical exercise while also reducing depressive levels among older adults. Lastly, there was a positive correlation between DI and diet (B = 0.030, SE = 0.003, *P* = 0.043), exercise (B = 0.030, SE = 0.018, *P* = 0.022), as well as smoking and drinking (B = 0.029, SE = 0.004, *P* = 0.021). Greater exposure to DI was associated with improved dietary habits and increased physical exercise but also higher rates of smoking. In conclusion, digital use, especially DE, DC, and DI, can significantly contribute to improving Chinese older adults’ diet, sleep, exercise, and depression levels. Digital use can enhance older adults’ online experience and improve their happiness levels, and it may also influence the formation of healthy eating habits and daily exercise patterns [39], boost self-efficacy and alleviate anxiety.

**Table 6:** The effect of TDU on the lifestyle of Chinese older adults

Influence Path	B	SE	P
DE→Diet	0.053^*^	0.007	0.019
DE→Sleep	0.222^**^	0.007	0.001
DE→Exercise	0.121^***^	0.045	< 0.001
DE→Smoking and drinking	−0.007	0.009	0.726
DE→Depression	−0.055^**^	0.009	0.008
DC→Diet	0.057^**^	0.010	0.006
DC→Sleep	0.133	0.010	0.059
DC→Exercise	0.109^***^	0.058	< 0.001
DC→Smoking and drinking	−0.021	0.012	0.230
DC→Depression	−0.056^**^	0.012	0.004
DI→Diet	0.030^*^	0.003	0.043
DI→Sleep	0.001	0.004	0.983
DI→Exercise	0.030^*^	0.018	0.022
DI→Smoking and drinking	0.029^*^	0.004	0.021
DI→Depression	−0.017	0.004	0.213
DL→Diet	−0.008	0.066	0.690
DL→Sleep	0.062	0.068	0.345
DL→Exercise	0.032	0.395	0.059
DL→Smoking and drinking	−0.006	0.082	0.722
DL→Depression	−0.000	0.082	0.979

*P < 0.05, **P < 0.01, ***P < 0.001

## Discussion

From the perspective of digital use breadth, Chinese older adults who are male, reside in urban areas, possess higher levels of education, and receive medical benefits exhibited a greater propensity to possess a great number of TDU, which is in part related to socioeconomic status and Internet use autonomy of older adults [54, 55]. Furthermore, even among Chinese older adults who possess digital devices, there existed a small proportion who have either never utilized or only used one of mentioned TDU. This indicated that despite owning devices, some Chinese older adults still face challenges related to insufficient digital literacy. To address this issue and bridge the digital divide among Chinese older adults, it is crucial to enhance their digital literacy [56]. From the perspective of FDU, Chinese older adults tended to exhibit lower levels of engagement, particularly in the domains of DC and DL. Even if they possess smart devices, they may still be reluctant or unable to use specific functions such as consumption, learning and social interaction on these devices [57], thereby further widening their estrangement from modern society [58] and exacerbating their digital divide. From the perspective of TDU, the younger Chinese older adults exhibited a greater propensity for engaging in DE (short video) and DC. Urban seniors were more likely to participate in DE (online game) while also engaging in DC and DI. Conversely, rural older adults tended to favor DE (short video) pursuits. From the perspective of the basic situation of lifestyle, the lifestyle differences between Chinese older adults who use digital devices and those who do not were significant. For example, Chinese older adults who use digital devices consumed more protein, had higher protein intake, slept more health adequately, and exercised more frequently.

Further analysis was conducted to explore the correlation between digital use and lifestyles among Chinese older adults. In terms of FDU, it had a substantial positive impact on the lifestyle of Chinese older adults. The findings from the structural equation model indicated that FDU had a significant positive effect on diet, exercise, smoking and drinking, as well as reduced depression among Chinese older adults, thereby effectively assisting them in cultivating a healthy life across various aspects including diet, exercise, sleep, smoking and drinking, as well as depression. In relation to TDU, a noteworthy positive correlation was evident, specifically between DE/DC and lifestyles. For instance, the utilization of DE could enhance healthy dietary intake, sleep quality, exercise level and alleviate depression among Chinese older adults. Similarly, DC had the potential in improving dietary habits along with exercise levels and reducing depression among Chinese older adults. Regarding DI’s impact on the lifestyle of Chinese older adults, it found that DI positively affected healthy dietary and exercise habits.

This research is subject to several limitations. Firstly, the indicator settings of certain variables require further refinement and clarification to enhance the verifiability of the conclusions. For example, while this paper considers TDU such as DE, DC, DI and DL based on the “Basic Knowledge and Skills of Health Literacy of Chinese Citizens”, it should also take into account other aspects such as digital public service and digital travel. Secondly, this study solely focuses on discussing the relationship between variables. Therefore, investigating the mechanisms and pathways that influence this relationship is necessary. The potential mediating role of personal emotions [59], family factors [60], living environment [61] and other factors should be investigated as they may significantly influence the association between digital use and Chinese older adults’ lifestyle. Thirdly, when examining the relationship between variables, additional influencing factors such as personal income [60], support from children [62] should be considered to further validate the applicability of these conclusions.

Despite these limitations, this study retains some informative value. The “UN Decade of Healthy Ageing: According to the Plan of Action (2021–2030)” mentioned that the opportunity to live longer depends largely on healthy ageing, which necessitates excellent physical and mental health as well as positive social interactions among older adults, which can be partially met by digital empowerment [63]. The enlightening significance of this paper lies in two aspects. Firstly, appropriate online activities can indeed promote a certain extent of healthy lifestyles among Chinese older adults. However, numerous Chinese older adults continue to face a challenging digital divide, particularly due to insufficient digital use skills. This calls for the development of a comprehensive social grand synergy model that promotes the enhancement of Chinese older adults’ digital skills. It is crucial for governments to prioritize optimizing Internet access conditions and fostering a respectful social environment as prerequisites for their engagement in online activities. Implementation a mobile access plan specifically for Chinese older adults is essential, providing subsidies for smartphones and mobile networks to those who are unable to afford digital technologies on their own. Simultaneously, there should be an improvement in network conditions as the government actively collaborates with network providers to introduce complimentary internet access plans tailored towards Chinese older adults’ needs. In addition, businesses should also focus on the development of age-appropriate products and encourage family members to provide “digital feedback” to Chinese older adults, so as to improve their propensity to use the Internet. Secondly, the excessive use of digital entertainment can partially contribute to unhealthy lifestyles among Chinese older adults. Consequently, it is imperative for them to proactively prevent Internet addiction. Moreover, a collaborative effort among the government, businesses, families and older adults themselves is essential in mitigating the risks associated with Internet addiction. Specifically, the government should strengthen the management and supervision of Internet enterprises and various media platforms. Simultaneously, Internet enterprises should promptly introduce age-appropriate products tailored to suit the characteristics of Chinese older adults. Chinese family members should pay attention to communication with older adults while assuming a role in digital reverse mentoring. More importantly, Chinese older adults themselves must actively adjust their behaviors, effectively manage their online time and avoid succumbing to Internet addiction.

## Conclusions

This paper utilized data from the 2020 Chinese Family Panel Studies (CFPS) to investigate whether digital use leads to Chinese older adults impacting their healthy lifestyles. The study’s findings suggested that overall digital use had a significant positive effect on Chinese older adults’ lifestyles, with digital use making Chinese older adults more diligent in maintaining a healthier lifestyle. To varying degrees, FDU and TDU also partially affected healthy lifestyles, including diet, exercise, sleep, smoking and drinking, and depression.

## Data Availability

All data generated or analysed during this study are included in this published article.

## Abbreviations

FDU: frequency of digital use
TDU: types of digital use
DE: digital entertainment
DC: digital consumption
DI: digital social interaction
DL: digital learning
CFPS: Chinese Family Panel Studies

## References

[1] China Internet Network Information Center. The 52nd Statistical Report on China’s Internet Development. 2023. https://www.cnnic.net.cn/n4/2023/0828/c88-10829.html. Accessed 5 Dec 2023.

[2] Pender NJ, Murdaugh CL, Parsons MA (2010). Health Promotion in nursing practice. Nurs Standard.

[3] National Health Commission of PRC. Basic Knowledge and Skills of Health Literacy of Chinese Citizens. 2016. http://www.nhc.gov.cn/xcs/s3581/201601/e02729e6565a47fea0487a212612705b.shtml. Accessed 5 Dec 2023.

[4] Wang S, Wu Y, Ungvari GS (2017). Sleep duration and its association with demographics, lifestyle factors, poor mental health and chronic diseases in older Chinese adults. Psychiatry Res.

[5] Stockwell S, Schofield P, Fisher A (2019). Digital behavior change interventions to promote physical activity and/or reduce sedentary behavior in older adults: a systematic review and meta-analysis. Exp Gerontol.

[6] Ding X, Yuan L, Zhou Y. Internet access and older adults’ health: evidence from China. China Econ Rev. 2023;82. 10.1016/j.chieco.2023.102047.

[7] Guo E, Li J, Luo L (2022). The effect and mechanism of internet use on the physical health of the older people-empirical analysis based on CFPS. Front Public Health.

[8] Chang SJ, Yang E, Lee KE (2021). Internet health information education for older adults: a pilot study. Geriatr Nurs.

[9] Rosell J, Vergés A, Miranda-Castillo C (2022). Predictors, types of internet use, and the Psychological Well-Being of older adults: a Comprehensive Model. J Gerontol B Psychol Sci Soc Sci.

[10] Quintana D, Cervantes A, Sáez Y (2018). Internet use and Psychological Well-being at Advanced Age: evidence from the English Longitudinal Study of Aging. Int J Environ Res Public Health.

[11] Marques LP, Schneider IJ, d’Orsi E. Quality of life and its association with work, the internet, participation in groups and physical activity among the elderly from the EpiFloripa survey, Florianópolis, Santa Catarina State, Brazil. Cad Saude Publica. 2016;32(12). 10.1590/0102-311X00143615. 10.1590/0102-311X00143615 28001209

[12] Zhang H, Wang H, Yan H (2021). Impact of internet use on Mental Health among Elderly individuals: a difference-in-differences study based on 2016–2018 CFPS Data. Int J Environ Res Public Health.

[13] Sun K, Zhou J. Understanding the impacts of internet use on senior citizens’ Social Participation in China: evidence from Longitudinal Panel Data. Telematics Inform. 2021;59. 10.1016/j.tele.2021.101566.

[14] Heo J, Chun S, Lee S, Cyberpsychology (2015). Behav Social Netw.

[15] Wallinheimo AS, Evans SL (2021). More frequent internet use during the COVID-19 Pandemic associates with enhanced quality of Life and Lower Depression scores in Middle-aged and older adults. Healthc (Basel).

[16] Feng Z, Cramm JM, Nieboer AP (2020). Social Participation is an Important Health Behaviour for Health and Quality of Life among chronically Ill Older Chinese people. BMC Geriatr.

[17] Quittschalle J, Stein J, Luppa M, et al. Internet use in Old Age: results of a German Population-Representative Survey. J Med Internet Res. 2020;22(11). 10.2196/15543. 10.2196/15543 PMC7685698 33226351

[18] Cui GH, Li SJ, Yin YT (2021). The relationship among social capital, eHealth literacy and health behaviours in Chinese elderly people: a cross-sectional study. BMC Public Health.

[19] Mozafar Saadati H, Mirzaei H, Okhovat B, et al. Association between internet addiction and loneliness across the world: a meta-analysis and systematic review. SSM Popul Health. 2021;16. 10.1016/j.ssmph.2021.100948. 10.1016/j.ssmph.2021.100948 PMC8563346 34754896

[20] Xiao W, Peng J, Liao S. Exploring the associations between Social Media Addiction and Depression: Attentional Bias as a Mediator and Socio-Emotional competence as a moderator. Int J Environ Res Public Health. 2022;19(20). 10.3390/ijerph192013496. 10.3390/ijerph192013496 PMC9602543 36294077

[21] Sun X, Yan W, Zhou H (2020). Internet use and need for digital health technology among the elderly: a cross-sectional survey in China. BMC Public Health.

[22] Firth J, Solmi M, Wootton RE (2020). A meta-review of lifestyle psychiatry: the role of exercise, smoking, diet and sleep in the prevention and treatment of mental disorders. World Psychiatry.

[23] Ren Y, Lin C, Zhou Q, et al. Effectiveness of virtual reality games in improving physical function, balance and reducing falls in balance-impaired older adults: a systematic review and meta-analysis. Arch Gerontol Geriatr. 2023;108. 10.1016/j.archger.2023.104924. 10.1016/j.archger.2023.104924 36680968

[24] Clemenson GD, Stark SM, Rutledge SM, et al. Enriching hippocampal memory function in older adults through video games. Behav Brain Res. 2020;390. 10.1016/j.bbr.2020.112667. 10.1016/j.bbr.2020.112667 PMC7286064 32439346

[25] Rybaczewska M, Sparks L (2022). Ageing consumers and e-commerce activities. Ageing Soc.

[26] Liu Q, Pan H, Wu Y. Migration status, internet use, and social participation among middle-aged and older adults in China: consequences for Depression. Int J Environ Res Public Health. 2020;17(16). 10.3390/ijerph17166007. 10.3390/ijerph17166007 PMC7459605 32824867

[27] Schehl B, Joerg L, Sugumaran V (2019). Understanding differentiated internet use in older adults: a study of Informational, Social, and Instrumental Online activities. Comput Hum Behav.

[28] Hengeveld LM, de Goede J, Afman LA (2022). Health effects of increasing protein intake above the current Population reference intake in older adults: a systematic review of the Health Council of the Netherlands. Adv Nutr.

[29] López-González L, Becerra-Tomás N, Babio N (2021). Variety in fruits and vegetables, diet quality and lifestyle in an older adult mediterranean population. Clin Nutr.

[30] Rie A, Kumi M, Misa S, et al. Drivers’ lunch break, health, and work performance: a study on Japanese drivers at a Courier Company who skip lunch. J Nutr Educ Behav. 2017;49. 10.1016/j.jneb.2017.05.335.

[31] Lin CL, Tsai YH, Yeh MC (2016). Associations between sleep duration and type 2 diabetes in Taiwanese adults: a population-based study. J Formos Med Assoc.

[32] Zhou B, Jiang C, Zhang W (2023). Association of sleep duration and napping with stroke mortality in older Chinese: a 14-year prospective cohort study of the Guangzhou Biobank Cohort study. Sleep Med.

[33] Li M, Ren Y (2023). Relationship among physical exercise, social support and sense of coherence in rural left-behind children. J Psychiatr Res.

[34] Mora JC, Valencia WM (2018). Exercise and older adults. Clin Geriatr Med.

[35] Mackenbach JP, Damhuis RA, Been JV. De Gezondheidseffecten Van Roken. The effects of smoking on health: growth of knowledge reveals even grimmer picture. Ned Tijdschr Geneeskd. 2017;160. 28098043

[36] Payne ME, Porter Starr KN, Orenduff M (2018). Quality of Life and Mental Health in older adults with obesity and Frailty: associations with a weight loss intervention. J Nutr Health Aging.

[37] Wang YW, Dai XH, Liu Y (2020). Does education affect physical health? - - -Based on data from the China Family Tracking Survey (CFPS). J Cent China Normal Univ (Humanities Social Sci Edition).

[38] Lu JH, Wei XD, Research on the impact of elderly people’s network participation on their social. Popul J. 2023;45(1):54–67. 10.16405/j.cnki.1004-129X.2023.01.007. Trust—A Test Based on CGSS Data of 2018.

[39] Shi J, Liu M, Fu G, et al. Internet use among older adults: determinants of usage and impacts on individuals’ well-being. Comput Hum Behav. 2023;139. 10.1016/j.chb.2022.107538.

[40] Li L, Jin G, Guo Y (2023). Internet access, support, usage divides, and depressive symptoms among older adults in China: a nationally representative cross-sectional study. J Affect Disord.

[41] Pu X, Wang Y, Zhang W, et al. Can basic medical insurance reduce elderly family income inequality in China? Front Public Health. 2022;10. 10.3389/fpubh.2022.838733. 10.3389/fpubh.2022.838733 PMC8885622 35242735

[42] Li L, Cai J, Chen W. How does transport development contribute to rural income in China? Evidence from county-level analysis using structural equation model. Travel Behav Soc. 2024;34. 10.1016/j.tbs.2023.100708.

[43] Jöerg E, Mikko R. Recent developments in PLS. Communications of the association for information systems. 2023;52:663–91. 10.17705/1CAIS.05229.

[44] Schuberth F, Hubona G, Roemer E. The choice of structural equation modeling technique matters: a commentary on dash and Paul (2021). Technol Forecast Soc Chang. 2023;194. 10.1016/j.techfore.2023.122665.

[45] Susanty S, Sarasmita MA, Sudarma IW (2023). Animated video development COVID-19 prevention and management for anxiety among older adults in Indonesia. Geriatr Nurs.

[46] Gyasi RM, Phillips DR, Asante F, Boateng S (2021). Physical activity and predictors of loneliness in community-dwelling older adults: the role of social connectedness. Geriatr Nurs.

[47] Wagner N, Hassanein K, Head M (2014). The impact of age on website usability. Comput Hum Behav.

[48] Park S, Kim B (2020). Readiness for utilizing digital intervention: patterns of internet use among older adults with diabetes. Prim Care Diabetes.

[49] Sun Y, Weng FZ, Yang GC (2023). The Effect of Physical Exercise on Sleep Quality———The mediating role of Mobile phone addiction. Bull Sport Sci Technol.

[50] Wolfe A, Policy, Politics (2001). Nurs Pract.

[51] Barbosa NB, Franz R, Judges R (2019). Can Digital Technology Enhance Social Connectedness among older adults? A feasibility study. J Appl Gerontol.

[52] Stickley A, Koyanagi A, Koposov R (2014). Loneliness and Health Risk Behaviours among Russian and U.S. adolescents: a cross-sectional study. BMC Public Health.

[53] Chen YR, Schulz PJ. The effect of information communication technology interventions on reducing social isolation in the elderly: a systematic review. J Med Internet Res. 2016;18(1). 10.2196/jmir.4596. 10.2196/jmir.4596 PMC4751336 26822073

[54] Hargittai E, Piper AM, Morris MR (2019). From Internet Access to Internet skills: Digital Inequality among older adults. Univ Access Inf Soc.

[55] Cresci M, Yarandi H, Morrell R (2010). Pro-nets Versus No-Nets: differences in urban older adults’ predilections for internet use. Educ Gerontol.

[56] Lee H, Lim JA, Nam HK. Effect of a digital literacy program on older adults’ digital social behavior: a quasi-experimental study. Int J Environ Res Public Health. 2022;19(19). 10.3390/ijerph191912404. 10.3390/ijerph191912404 PMC9564917 36231707

[57] Ma T, Zhang S, Zhu S (2022). The new role of nursing in digital inclusion: reflections on smartphone use and willingness to increase digital skills among Chinese older adults. Geriatr Nurs.

[58] Antonucci TC, Ajrouch KJ, Birditt KS (2014). The convoy model: explaining social relations from a multidisciplinary perspective. Gerontologist.

[59] Uchino BN, Rook KS (2020). Emotions, relationships, health and illness into old age. Maturitas.

[60] Mitra S, Gao Q, Chen W, et al. Health, work, and income among middle-aged and older adults: a panel analysis for China. J Econ Ageing. 2020;17. 10.1016/j.jeoa.2020.100255.

[61] Liu Y, Dijst M, Faber J (2017). Healthy urban living: residential environment and health of older adults in Shanghai. Health Place.

[62] Wu Y, Dong K, Bai R (2023). The relationship between intergenerational financial support and depressive symptoms among older adults: evidence from China Health and Retirement Longitudinal Study, 2011–2018. J Affect Disord.

[63] United Nations. Decade of Healthy Ageing. UN Decade of Healthy Ageing: Plan of Action. 2020:2021–2030. https://www.who.int/docs/default-source/decade-of-healthy-ageing/final-decade-proposal/decade-proposal-final-apr2020-en.pdf?sfvrsn=73137ef_4. Accessed 5 Dec 2023.

